# Sea-level rise will likely accelerate rock coast cliff retreat rates

**DOI:** 10.1038/s41467-022-34386-3

**Published:** 2022-11-18

**Authors:** Jennifer R. Shadrick, Dylan H. Rood, Martin D. Hurst, Matthew D. Piggott, Bethany G. Hebditch, Alexander J. Seal, Klaus M. Wilcken

**Affiliations:** 1 Department of Earth Science and Engineering, Imperial College London, London, UK; 2 School of Geographical and Earth Sciences, University of Glasgow, Glasgow, UK; 3 Centre for Accelerator Science, Australian Nuclear Science and Technology Organization (ANSTO), Lucas Heights, Sydney, NSW Australia

**Keywords:** Climate-change impacts, Natural hazards, Geomorphology

## Abstract

Coastal response to anthropogenic climate change is of central importance to the infrastructure and inhabitants in these areas. Despite being globally ubiquitous, the stability of rock coasts has been largely neglected, and the expected acceleration of cliff erosion following sea-level rise has not been tested with empirical data, until now. We have optimised a coastal evolution model to topographic and cosmogenic radionuclide data to quantify cliff retreat rates for the past 8000 years and forecast rates for the next century. Here we show that rates of cliff retreat will increase by up to an order of magnitude by 2100 according to current predictions of sea-level rise: an increase much greater than previously predicted. This study challenges conventional coastal management practices by revealing that even historically stable rock coasts are highly sensitive to sea-level rise and should be included in future planning for global climate change response.

## Abstract (web summary)

Results forecast that cliff retreat rates will increase by up to an order of magnitude by 2100 according to current predictions of sea-level rise, and reveal that even historically stable rock coasts are highly sensitive to sea-level rise.

## Introduction

Cliff erosion on rock coasts is manifestly an immediate hazard to human lives, property and infrastructure, which will only intensify as coastal urbanisation continues to increase rapidly^1^. Climate change is accelerating sea-level rise (SLR); global-mean SLR is predicted to exceed 1 m by 2100 unless greenhouse gas emissions are reduced^2^. Efforts to anticipate future coastal erosion rates and related hazards have largely focused on soft, erodible coastlines^3^ and have until recently neglected rock coasts^4^. Rock coasts make up >50% of global coastlines^5–8^ and are themselves evidence of ongoing erosion.

SLR is expected to result in wave energy reaching further inshore to attack coastal cliffs and trigger increased rates of cliff retreat, where cliff retreat refers to the landward movement of the cliff top edge^9^. Relatively few studies have endeavoured to capture such a response and make predictions of cliff retreat rates using a variety of coastal evolution models. For example, the Trenhaile model^10^ is a well-established process-based model that represents the evolution of an across-shore rock coast profile and cliff retreat is principally controlled by broken wave transformation across a shore platform. This model suggests that rising sea-level will trigger accelerated rates of cliff retreat and the greatest proportional increases will be seen at historically slower eroding coastlines^11^. However, these predictions are entirely theoretical, and results were not calibrated to site-specific data. Another prominent rock coast evolution model is the Soft Cliff And Platform Erosion (SCAPE) model^12^ that has been used to derive a simplified expression between cliff retreat rate and the relative change in the rate of SLR, which permits predictions of future cliff retreat rates^13–15^. Nevertheless, the SCAPE model is only appropriate for soft rock coastlines. Furthermore, the SCAPE-derived expression relating cliff retreat rate to SLR is limited to rock coasts under equilibrium conditions, i.e., only constant SLR^13^. For these reasons, the SCAPE model is not best suited for investigating cliff retreat response to future scenarios of accelerated SLR at hard rock coastlines. Other previous studies have often used 1-D models to make cliff retreat rate predictions that simplify processes to relate wave force and SLR to observed rates of cliff retreat^9,15–18^. These models are useful for considering large spatial and temporal scales, but their predictions rely heavily on historical observations of cliff retreat^9^. Historical observations are prone to large uncertainties not only because of the inaccuracy of old maps that must be used to reconstruct past cliff positions^19^, but also the unreliability of short cliff retreat records^20^. Furthermore, contrary to theory, a recent site-specific study found no link between past SLR and cliff retreat rates^21^. Consequently, significant uncertainty still surrounds the response of cliffed coasts to future SLR^15^, especially at sites with hard rock types over prehistoric timescales, and remains a central challenge faced by coastal engineers, managers, and policy makers^4^.

Further difficulty involved in the prediction of rock coast response to climate change exists because of the complex interplay between the relevant processes, and the fact that these processes traverse a range of temporal scales. Foremost, erosion of rock coasts is conditional on the type and structure of rock present at the coast^20,22–24^, which implies that rock weathering is an important component of cliff retreat^25–27^. SLR, tides and tidal currents, wave energy and the state and variability of air and water temperature work in conjunction to mediate the energy delivery to cliffs and influence the efficacy of erosion and transport processes^28^.

Previous studies of selective erosion processes, however, often cover only short time periods relative to the timescales over which rock coasts change. The longest observational record of shore platform vertical erosion rates of ~0.5–1.2 mm y^−1^ spans only 43 years^29^ and evidence of past cliff retreat obtained from historical records covers only ~150 years^30,31^ and more recently up to 400 years^32^. To reiterate, these historic records of cliff retreat, with associated large uncertainties, are central to many predictive models that explore cliff retreat response to SLR. Cliff erosion is intrinsically episodic, and if the period between erosion events (e.g., cliff collapse) is longer than the length of the observational record, erosion risk can be underestimated and even appear negligible. Furthermore, projected rates of SLR to 2100 are unprecedented during this period of historic observation, which makes future prediction, based on extrapolations of observed, short-term records, problematic^15^. Long-term, prehistoric records of past erosion at rock coasts that are representative of long-term trends and include when past rates of SLR were comparable to projected future rates, are, therefore, vital to our ability to anticipate future change in response to SLR^1,4^. Longer records of past erosion at rock coasts that are representative of long-term trends and include when past rates of SLR were comparable to projected rates are, therefore, vital to our ability to couple future change in response to SLR^1,4^.

Until now, reconstructing long-term, pre-historic records of coastal change was a challenge because rock coasts, by their nature, leave scant evidence of their former state. However, cosmogenic radionuclides (CRNs, rare isotopes that build up in situ in rock near to the Earth’s surface when exposed to cosmic rays) have transformed our ability to investigate coastal cliff retreat over centennial to millennial timescales^1,4,33^. Concentrations of rare nuclides (e.g., ^10^Be) measured in a rock sample reflect how long a rock has been exposed (its geomorphic age), or how rapidly it has been unveiled (its erosion rate)^34^. On rock coasts, the erosion of cliffs and the resultant landward retreat of the coast leaves, in its wake, an intertidal shore platform. Spatial patterns of CRN concentrations across shore platforms complexly reflect the age and rate of that cliff retreat^33^. Several recent studies have used CRNs to quantify long-term cliff retreat rates^1,21,33,35,36^ and to explore the antiquity of shore platforms^37^. These studies, however, relied on relatively simple geometric models of rock coast evolution that were unable to capture transient long-term cliff retreat rates or represent physical erosion processes.

In order to advance earlier studies, we apply our new methodology^38^. In this methodology, we use multi-objective optimisation to calibrate a process-based coastal evolution model to both high-precision ^10^Be concentration measurements and observed coastal topography. Unlike previous work, by optimising the coupled Matsumoto et al.^39^ rock coast evolution model and CRN production model^40^ we can derive transient long-term cliff retreat rates for real rock coast sites and represent physical erosion processes. Our model optimisation focuses on parameters that control the efficacy of wave erosion and intertidal weathering relative to the resistance of the bedrock^41^. Our model inversions minimise discrepancies between modelled and observed ^10^Be concentrations and rock coast topography to reveal the most likely history of rock coast evolution for the past 8000 years^38^. These long-term, past cliff retreat rates are therefore informed with empirical data, are independent of historical records of cliff retreat, and encompass a time where past rates of SLR are comparable to projected future rates of accelerated SLR^42^. After optimising the model to the measured ^10^Be concentrations and topography, simulations are then coupled to UKCP18 future SLR predictions^43^ to anticipate future cliff retreat rates in the face of climate change (Methods).

## Results

### Study site background

**Fig. 1:**
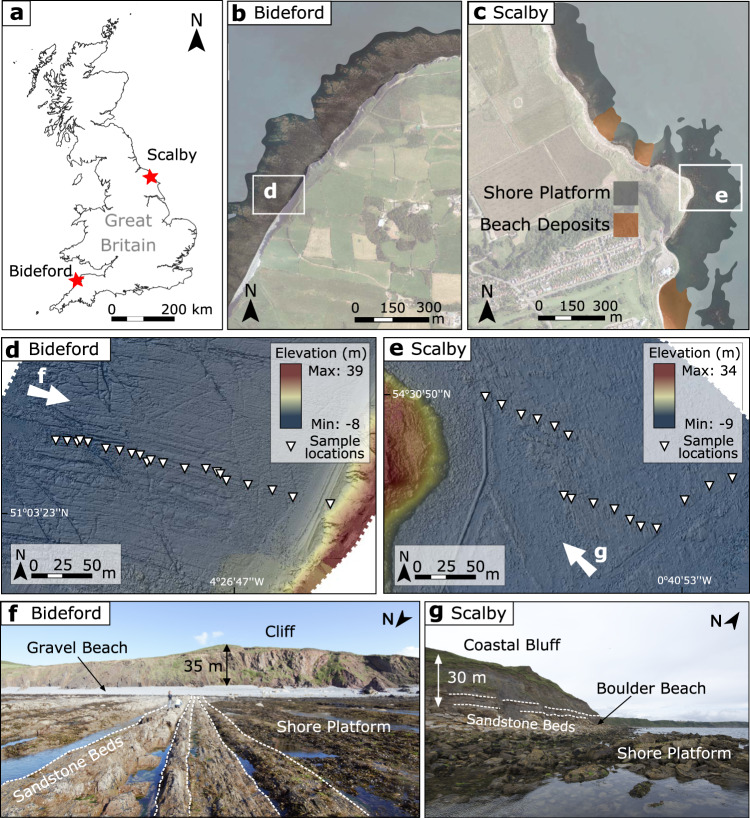
Study sites. **a** Map of Great Britain with sites located. **b** Bideford sample site location with aerial imagery and location of shore platform shown. **c** Scalby sample site location with aerial imagery and location of shore platform shown. **d** Bideford shaded relief digital surface model (DSM) and cosmogenic radionuclide (CRN) sample locations. Look direction for panel **f** shown with arrow. **e** Scalby shaded relief DSM and CRN sample locations. Look direction for panel **g** shown with arrow. **f** Bideford field photo with features identified including a near-vertical cliff. **g** Scalby field photo with features identified including a gently sloping coastal bluff.

We apply our new modelling methodology to two sites in the United Kingdom (UK), at Bideford (north Devon) and Scalby (Yorkshire) where we reconstruct long-term cliff retreat rates using CRN analysis and digital surface model (DSM) acquisition (Fig. 1a). SLR histories are site-specific and provided by a glacial isostatic adjustment (GIA) model^44^. Both sites have similar SLR histories that show a declining rate of SLR across the late Holocene, where sea level 8000 years BP (Before Present, where present is defined as the year 2000 CE) was ~16 m lower.

### Measured cosmogenic radionuclide concentrations

**Fig. 2:**
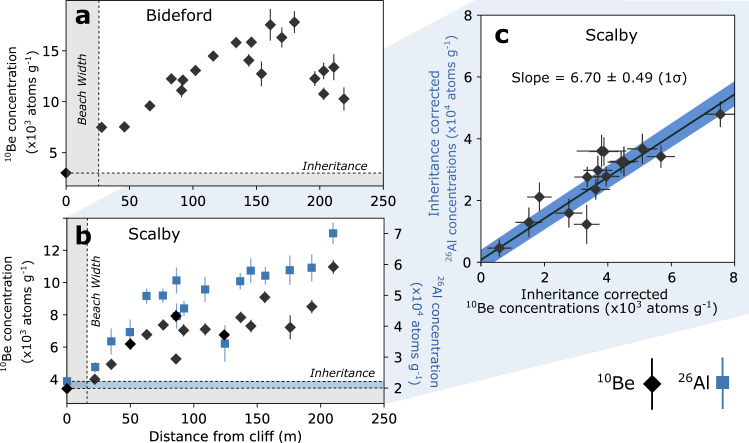
Measured cosmogenic radionuclide (CRN) concentrations. **a** Measured ^10^Be concentrations at Bideford with distance from the cliff base (1$\sigma$). **b** Measured ^10^Be and ^26^Al concentrations at Scalby with distance from the cliff base (1$\sigma$). A shielded cliff sample was used to correct for any inherited CRNs present in the exposed platform rock samples to ensure that concentrations of ^10^Be and ^26^Al solely reflect the exposure time due to cliff retreat (Methods). **c** Inheritance-corrected ^10^Be and ^26^Al concentrations for Scalby. Slope of regression line = 6.70 ± 0.49 (1$\sigma$), and is comparable to the production rate ratio in quartz of ~6.75 ± 0.50 (1$\sigma$)^49^. Intercept = 795 ± 3301 ^26^Al atoms g^−1^ (1$\sigma$). Concentration are corrected for chemistry background using process blank samples and inherited CRN concentrations using shielded cliff samples (at distance 0 m) with errors propagated in quadrature.

At both sites, we measured CRN concentrations in rock samples collected from the shore platform along a transect perpendicular to the coastline (Fig. 1d, e). At Bideford, the relatively low and humped ^10^Be concentrations profile is evidence of a shore platform eroded in the Holocene^1,33,40^ (Fig. 2). Furthermore, observed and documented raised marine terraces in the cliffs of the north Devon coast suggest sea level at the previous interglacial stage, or older, was at a considerably greater elevation than it is presently^45–47^. At the previous interglacial stage, Marine Isotope Stage (MIS) 5e (~130–115 k years BP)^48^, global-mean sea level (GMSL) was ~4–6 m higher than present-day sea level and could correspond to the raised marine terraces observed at Bideford. Thus, it seems unlikely that the modern shore platform is a reoccupation of topography eroded during this previous relative sea-level high. Furthermore, GMSL peaked at −8.5 ± 4.6 m during MIS 5a (~80 k years BP) and −9.4 ± 5.3 m during MIS 5c (~100 k years BP)^48^. These elevations are within the range of relative sea-level elevations for the past 8000 years. If the present-day intertidal platform was reoccupied from these two sea-level high stands, we would expect to see a sharp step in ^10^Be concentrations^33^, which we do not see at either Bideford or Scalby. Therefore, we suggest it is most likely that the intertidal shore platform at Bideford and Scalby formed during the Holocene, rather than inherited topography from a previous relative sea-level high stand.

In addition, we have used multi-nuclide CRN analyses to further investigate the potentially complex, exposure history of a shore platform inherited from a previous interglacial period. At Scalby, we measured ^10^Be and ^26^Al, to further test the hypothesis that these rock platforms were formed during the present interglacial period of sea level rise. Our multi-nuclide analyses at Scalby indicate a ^26^Al/^10^Be of 6.70 ± 0.49 (1$\sigma$) (Fig. 2), which is consistent with the surface production rate ratio in quartz of ~6.75 ± 0.50 (1$\sigma$)^49^. The combination of these ^26^Al/^10^Be results as well as the low absolute ^10^Be and ^26^Al concentrations provide compelling evidence that the platform at Scalby is entirely formed in the Holocene with no previous subaerial exposure, burial and/or reoccupation of the shore platform. We are confident, therefore, that our measured CRN concentrations can be reliably used to model long-term, Holocene cliff retreat history at these sites.

### Historical cliff retreat rates

To quantify historical cliff retreat rates, cliff positions were digitised from historical Ordnance Survey (OS) maps and recent aerial imagery using methods similar to previous studies^9,30^ (Methods). Both sites exhibit minimal historical cliff retreat with average rates for the past ~130 years of just 5.8 $\pm$ 4.0 cm yr^−1^ at Bideford and 5.9 $\pm$ 4.3 cm yr^−1^ at Scalby. However, even across small section of coastline, there is large variability in cliff retreat rates caused by the stochastic pattern of erosion in space and time (Supplementary Fig. 7, Supplementary Fig. 8). For a ~2 km section of coastline, historical cliff retreat rates range from 2 to 25 cm yr^−1^ at Bideford and Scalby.

### Past transient long-term cliff retreat rates

We used our multi-objective optimisation of the coupled rock coast evolution and CRN production model^38^ not only to reconstruct past transient long-term cliff retreat histories but also to identify the relative contributions of wave and weathering erosion processes for both sites. The model produced a good fit to both ^10^Be and topographic datasets (Supplementary Fig. 2). Importantly, for model results to match the corresponding measured ^10^Be and topographic datasets simultaneously, the optimised model parameters indicated negligible intertidal weathering and, therefore, the erosion processes at these sites are wave-dominated^38^. It is assumed that mechanisms such as wetting and drying, frost weathering, bioerosion and abrasion can be represented in our model by a single shape function informed by wetting and drying experiments^50^. Modelling platform weathering exclusively with patterns of wetting and drying is a common approach taken to quantify weathering efficacy at rock coasts^25,27,39^ because there is little empirical data to inform and isolate differing weathering activity broadly across different sites. However, our results are insensitive to weathering because negligible weathering is needed to match the ^10^Be concentrations at both sites. Importantly, different representations of weathering would therefore have minimal impact on the final cliff retreat results for these sites.

These best-fit model results were used to calculate transient cliff retreat rates for the past 8000 years. Cliff retreat rates at both sites show a decline across the late-Holocene that closely match the rate of SLR^38^ (Supplementary Fig. 3). For both sites, sea-level has constantly risen across the Holocene, whereas cliff retreat has constantly fallen (Supplementary Fig. 3). This correlation is consistent with the interpretation that cliff retreat is more sensitive to the rate of SLR than the absolute magnitude of SLR^15^. Present-day modelled cliff retreat rates are within the variability of historical cliff retreat rates at both sites, further corroborating our optimised model retreat rates. Our optimised model results^38^ provide compelling evidence that cliff erosion is dominated by waves and cliff retreat rates are strongly linked to the rate of SLR.

### Future cliff retreat rates

**Fig. 3:**
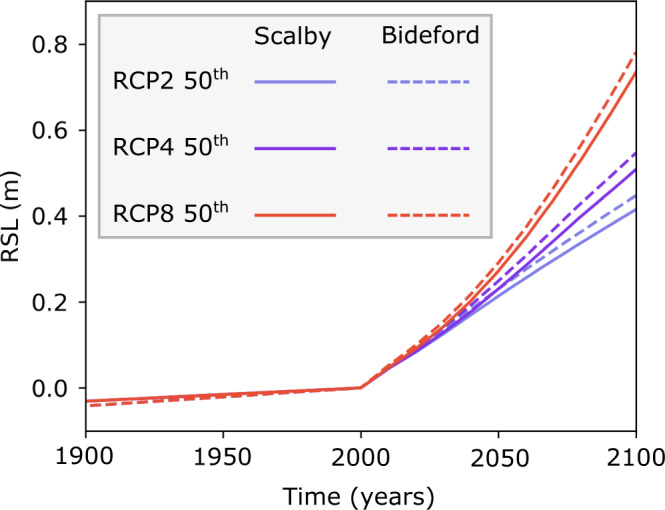
Relative Sea level (RSL) for the years 1900–2100 for Bideford and Scalby. Representative Concentration Pathways RCP2, RCP4 and RCP8 50th SLR projections for Scalby (solid line) and Bideford (dashed line). Sea-level rise (SLR) between the years 1900–2000 was informed with the glacial isostatic adjustment (GIA) model and SLR between the years 2000–2100 were informed with UKCP18 future SLR projections.

**Fig. 4:**
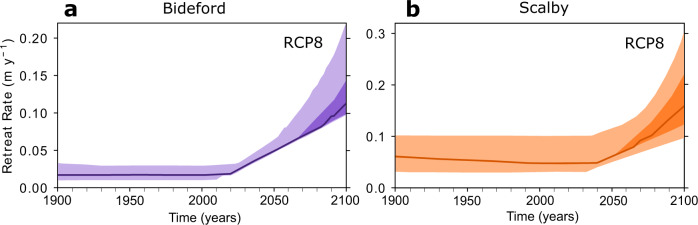
Model forecasts of future cliff retreat rates for Representative Concentration Pathway RCP8 future sea-level scenario. Bideford (**a**) and Scalby (**b**). Lightest-shaded areas combine model uncertainty with 5th–95th RCP8 sea-level percentiles. Medium-shaded areas combine best-fit model inputs with 5th–95th RCP8 sea-level percentiles. The darkest-shaded areas combine best-fit model inputs and 50th RCP8 percentile.

In order to determine the most likely forecast of cliff retreat rates into the future, we brought together the optimised, best-fit model parameters and the Representative Concentration Pathway 8.5 (RCP8.5) sea-level scenario, equivalent to the newly-defined Shared Socioeconomic Pathway 8.5 (SSP8.5) scenario^42^. The RCP8.5 is based on the current trajectory of greenhouse gas emissions^51^ and we have used this scenario to inform the most likely cliff retreat rate forecasts. After optimising the models to the measured data for the past (see the previous section), we reran the models with best-fit parameters to make forecasts of future cliff retreat rates. We ran the model with the optimised parameters starting at 8000 years BP to present-day informed by GIA relative sea-level for the past and continued the simulation to forecast to the year 2100 with the appended UKCP18 sea-level projections (Fig. 3). We did not model individual erosion events because they are inherently stochastic in nature and relate to uncertain local factors involved in triggering cliff failure, such as storm occurrence. Instead, we averaged episodic erosion events so that we could identify the long-term, future trend of cliff retreat rates. For Bideford, we forecast that the most likely trajectory of cliff retreat rates will be seven times faster by 2100 than the model rates for the last century (Fig. 4a). For the full range of future sea-level projections (RCP2–RCP8; including 5th–95th percentile probability ranges), we forecast that by 2100, cliff retreat rates at Bideford will increase between 5–13 times the model rates for the last century (Table S4). Similarly, at Scalby, we forecast that by 2100 the most likely trajectory of cliff retreat rates will increase by three times the model rates for the last century (Fig. 4b). The full range of future cliff retreat rate scenarios (RCP2–RCP8) at Scalby is also forecast to increase by 2–6 times the model rates for the last century with maximum cliff retreat rates of 30 cm y^−1^ (Table 1).

**Table 1:** Past cliff retreat rates and future Representative Concentration Pathway RCP2, RCP4 and RCP8, minimum, medium and maximum 2100 forecast cliff retreat rates

Past observed rates (m y^−1^)	Past modelled rates (m y^−1^)*	2100 cliff retreat rates (m y^−1^)**								
		RCP2			RCP4			RCP8		
		Min	Med	Max	Min	Med	Max	Min	Med	Max
**Bideford**										
0.058 ±0.041	0.017 +0.014/−0.009	0.080	0.099	0.160	0.099	0.099	0.171	0.099	0.112	0.215
**Scalby**										
0.059 ±0.043	0.054 +0.056/−0.024	0.098	0.125	0.194	0.098	0.126	0.217	0.098	0.159	0.300

*Past modelled retreat rates are the average rate between the years 1900–2000.

**Minimum and maximum forecast cliff retreat rates incorporate model uncertainty and RCP sea-level 5th–95th percentiles.

SLR starts to rapidly accelerate at 2000 calendar years at the start of the UKCP18 sea-level projections (Fig. 3). At Scalby, cliff retreat rates start to respond to these accelerations in SLR at ~2030 because of the faster cliff retreat rates modelled for the last century (Fig. 4). In contrast, at Bideford, cliff retreat rates respond faster and start at ~2020 (Fig. 4). As well as greater proportional increases in cliff retreat rates^11^, these results suggest that accelerations in cliff retreat rates will start sooner at sites with slower past cliff retreat rates for the last century.

## Discussion

Although the general distribution of ^10^Be concentrations and topography is well matched by the best-fit results at both sites, some data points deviate from the best-fit results (Expanded Data Fig. 8). Where we have been unable to capture individual data points with the best-fit model results, these deviations are likely a result of simplifications made in our model. These simplifications include, for example, homogeneous resistance across the shore platform that could explain the variability in offshore ^10^Be concentrations at Bideford (Expanded Data Fig. 6a). In reality, sandstone beds of varied thicknesses and grain sizes will erode at different rates and through different processes of erosion (e.g., Swirad et al.^52^). Heterogenous lithology could, therefore, also contribute to the variation in ^10^Be concentrations observed at Bideford. Nevertheless, Hurst et al.^40^ concludes that factors that influence the distribution of the ^10^Be concentration profile are all secondary to the primary control of cliff retreat rate. Furthermore, simplifications have to be made to modelled erosion processes to be applicable to millennial timescales^27,39^. Therefore, because we have captured the overall trend in both ^10^Be concentrations and topography, we can assume that our best-fit results are valid and able to inform our understanding of long-term cliff retreat.

Similar to previous studies, we also compare our forecast cliff retreat rates to observed, historical records. For a 1 m SLR at 2100 (equivalent to the RCP8 95th scenario), our most likely results show cliff retreat rates will be four times greater at Bideford and five times greater at Scalby compared to historical cliff retreat rates (Table 1). Furthermore, because our model results are independent of historical observations, we also compare our forecasts to past model rates for the last century. Comparisons of forecast cliff retreat rates to past model rates for the last century show even greater accelerations in cliff retreat rates; we forecast cliff retreat to increase by 13 times at Bideford and 6 times at Scalby for a 1 m SLR at 2100 (Table 1). Model results at Bideford suggest the long-term cliff retreat rates for the last century are much slower than the historical record suggests, which makes predictions of cliff retreat accelerations greater when compared to past model cliff retreat rates. Although past model rates for the last century are within the uncertainty of the historical record, these deviations between observed and model past cliff retreat rates could suggest that acceleration has already begun at Bideford.

In contrast to other model projections of cliff retreat rates, following a 1 m SLR, for both historical records and modelled rates for the past century, our forecasts are consistently higher than previous studies. Previous studies that include a wide range of lithological settings have quantified between 1.2 and 2.3 times increase in cliff retreat rates for 1 m SLR at 2100 compared to historical observations^9,14,16,53,54^. Our projections of cliff retreat rate accelerations following a 1 m SLR are, therefore, at least twice as much as previous studies, and up to an order of magnitude greater than previous predictions of cliff retreat accelerations. These projections are all site specific, so will vary according to specific parameter values used to calibrate the models to each rock coast site. Nevertheless, our forecasts of greater magnitude of acceleration in cliff retreat compared to previous studies are also likely caused by simplifications to previous models. These simplifications made to previous models were necessary in order to derive universal expressions relating cliff erosion to SLR. For example, Limber et al.^9^ did not consider the influence of tides and only an intermediate range of wave height decay rates. In contrast, here we consider site-specific tidal ranges based on tidal gauge data and a wider range of wave height decay rates informed by previous studies^27^. Inclusion of a tidal range duration distribution^10,27,39^ increases the vertical reach of wave erosion to enhance wave attack at the cliff base and increases the horizontal width of the surf zone across which breaking waves can erode the shore platform. Implementing a tidal range that is informed with measured data is therefore likely to increase cliff retreat rate predictions compared to models that do not consider tidal range. Furthermore, simplified 1-D models are less sensitive to extreme events^9^, therefore, it is not surprising that our process-based model, which is optimised to two distinctive, site-specific datasets, reveals a greater sensitivity of cliff retreat rates to SLR. Notably, cliff retreat rate sensitivity to SLR cannot be captured with historical observations and therefore has not been quantified and supported with empirical data at historically stable sites such as Bideford and Scalby until now.

Our cliff retreat rate projections can, however, be considered conservative estimates. Superimposed on SLR, climate change is also expected to cause increased storm frequency and intensity^55^. Although there are clear increasing trends of storm intensity in the North Atlantic since the 1980’s^56^, this is not consistent globally and large uncertainties obscure climate change impact on storms^56,57^. Although of less importance than increased SLR^11^, increased storminess is likely to accelerate erosion further at wave-dominated coastal sites^11,55^ and increase the speed of cliff retreat rates beyond our estimates in the future.

There is evidence of significant accelerations in historical cliff retreat rates at chalk cliff sites on the south coast on the UK. At these softer-rock sites, historical cliff retreat rates are ~4–16 times greater than the Holocene average^1^. Although no such acceleration in historical cliff retreat rates has been observed at our harder-rock sandstone sites, our forecasts suggest that despite their apparent stability during the historical period, with the anticipated SLR to 2100, these harder-rock coastlines will experience substantial increases in cliff retreat rates.

**Fig. 5:**
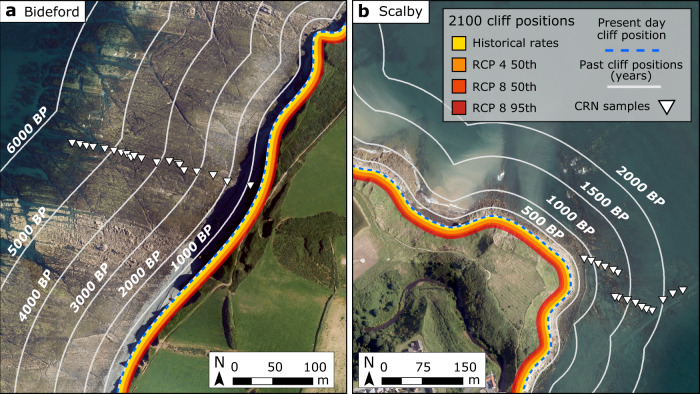
Year 2100 and past modelled cliff positions. Bideford site (**a**) and Scalby site (**b**). Future forecasts of cliff positions are shown for the year 2100 based on projections of observed historical retreat rates and Representative Concentration Pathways RCP4 50th, RCP8 50th and RCP8 95th future sea-level scenarios. Model forecasts of past cliff positions at 500-year intervals at Scalby and 1000-year intervals at Bideford.

Because cliff retreat events are stochastic in time and space, we cannot pinpoint exact future cliff positions or capture alongshore variability. Rather, by extrapolating the current cliff position using historical rates and future model forecasts of retreat rate increase, we can highlight areas of the coast that are vulnerable to cliff erosion in the next 100 years (Fig. 5). By 2100, optimised model results based on the most likely future sea-level scenario suggest that 11 m of cliff retreat will occur at Bideford and 16 m at Scalby. Comparisons between past and future forecasts of modelled cliff positions suggest that by the year 2100, the magnitude of cliff retreat during the next 100 years will likely be equivalent to the cliff retreat of the past ~500 years at Scalby and ~1000 years at Bideford (Fig. 5).

**Fig. 6:**
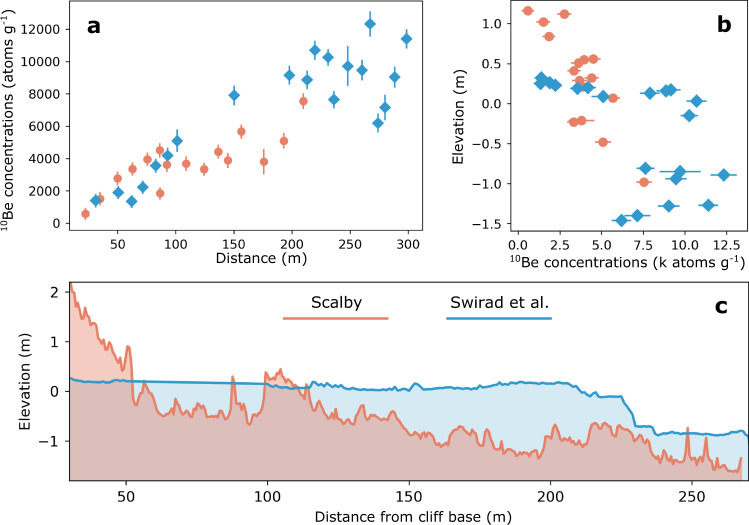
Comparisons between measured data at Scalby and Swirad et al.^21^ study site. Measured concentrations of ^10^Be (atoms g^−1^) as a function of distance from cliff base (m) (**a**). Measured ^10^Be concentrations (k atoms g^−1^) as a function of elevation (m) at Scalby and Swirad et al.^21^ study site (**b**). Across-shore topographic profile (m) (**c**).

Our results, which clearly demonstrate that cliff retreat rates reflect the rate of SLR at our sites, contrast with those of Swirad et al.^21^, who found no link between cliff retreat rates and the rate of SLR at another UK rock coast site. There are, however, two key distinctions between the different models used in the studies. First, Swirad et al.^21^ considered only model scenarios enforcing linear change in past retreat rates. Our model, by contrast, imposes no constraints on how retreat rate changes through time. This is because retreat rates emerge from the representation of physical processes^39^, and our model scenario that best matches the measured data is a non-linear decline in retreat rates that tracks the declining rate of SLR (Supplementary Fig. 3). Second, Swirad et al.^21^ uses a geometric model that reflects strong geological and stratigraphic control on cliff retreat rates and platform topography. Our process-based model does not account for geological heterogeneity as a control on shore platform topography because we do not observe similar geological control at our sites. Also, there are significant contrasts in the measured topography between both sites that highlight the clear stratigraphic control on the across-shore profile at the Swirad et al.^21^ site (Fig. 6c). A thick bed of resistant ironstone at the Swirad et al.^21^ site (Fig. 6c) has caused a near-horizontal across-shore profile with a step at ~230 m from the cliff base. In contrast, at our Scalby site, the shore platform has a semi-continuous, gently sloping profile with deviations caused by the location of different sandstone beds (Fig. 6c). Furthermore, the shore platform at Scalby is mostly at a lower elevation compared to the shore platform at the Swirad et al.^21^ site. The presence of a thick resistant stratigraphic bed has the potential to both limit shore platform lowering and prevent the location of the cliff-platform junction from tracking the elevation of SLR in a steady-state scenario. Considering the comparable SLR histories and tidal ranges at each site, the contrasts in topography alone suggest that, unlike at Scalby, the stratigraphic control evident at the site studied by Swirad et al.^21^ has dominated its long-term erosional history.

Stratigraphic control on the long-term erosional history at the Swirad et al.^21^ study site is further evidenced by the observed contrasts between the relationship of ^10^Be concentrations to topography at each site (Fig. 6a, b). Between ~100–200 m offshore from the cliff base, ^10^Be concentrations at Scalby are as much as ~4000 atoms g^−1^ lower compared to the site studied by Swirad et al.^21^ (Fig. 6a). Concentrations of ^10^Be are more comparable between ~0–100 m from the cliff base (Fig. 6a); however, significantly lower ^10^Be concentrations offshore at Scalby suggest that cliff retreat rates were faster in the past compared to the site studied by Swirad et al.^21^. This hypothesis is supported by our model findings that reveal cliff retreat rates were faster when rates of SLR were greater in the past at Scalby. Our transient cliff retreat rates reveal that the platform ~150–200 m offshore from the cliff was eroded between 1600–2000 years BP at Scalby. During this time, average cliff retreat rates were 0.125 m yr^−1^, which decrease to 0.054 m yr^−1^ at the present day as the rate of SLR declined (Supplementary Fig. 3). In contrast, for the site studied by Swirad et al.^21^, a constant cliff retreat rate history of 0.045 m yr^−1^ best matches their measured data. Furthermore, a comparison of ^10^Be concentrations to elevation (Fig. 6b) shows that greater concentrations are found at lower elevations at Scalby. In contrast, at the Swirad et al.^21^ study site, ^10^Be concentrations do not show the same relationship to elevation. At elevations −1–0 m, concentrations are considerably higher, which suggests that this part of the shore platform has been exposed for a longer duration of time at the site studied by Swirad et al.^21^. These differences in both measured topographic and ^10^Be data sets (Fig. 6) suggest that contrasting exposure histories occurred at each site, which is reflected by each best-fit model result. Most importantly, in this study, the geological structure does not mask the control of SLR on cliff retreat rates at Scalby and Bideford.

A previous study explored the sensitivity of cliff retreat rates, which were derived from a spectrum of coastal evolution models that represented varied drivers of cliff retreat, and their response to SLR^9^. This previous study concluded that the Trenhaile model^10^ is most sensitive to SLR due to the representation of exponential wave transformation across the surf zone. With exponential wave transformation, when SLR increases, the surf zone narrows and cliff retreat accelerates^9^. Wave force is similarly represented in our present model^39^. However, Limber et al.^9^ found that although the Trenhaile model was most sensitive to SLR, the magnitude of predicted cliff retreat rates was, in fact, intermediate to the four other models that were considered. Furthermore, the Trenhaile model^10^ only overpredicted cliff retreat rates once SLR was 2 m higher than present^9^. Furthermore, exponential wave height decay, as is employed in our model, is said to underestimate wave erosion, as infragravity waves that can reach the cliff base even when breaking far offshore are not represented^9^. The greatest increase in SLR we have considered (RCP8 95) reaches 1.10 m at Scalby and 1.15 m at Bideford at 2100. With 1–1.5 m SLR, cliff retreat rate forecasts made by the Trenhaile model align with the mean projections of the other models^9^. We can therefore be confident that our forecasts are credible, given our SLR scenarios and representation of wave erosion.

To conclude, our findings confirm a clear causal relationship between the rate of SLR and cliff retreat rate for two historically stable UK coastal sites. This relationship is evidenced by long-term cliff retreat rates that closely track the rate of SLR at both sites. Our model forecasts that the projected acceleration in the rate of SLR in response to climate change^2^ will accelerate cliff retreat. On this basis, by 2100, cliff retreat rates are forecast to accelerate by at least 3–7 times present-day rates and cliff positions are likely to retreat by at least 10–14 m at Bideford and 13–22 m at Scalby. These forecasts of cliff retreat acceleration are at least two times greater than previously estimated and further increase to as much as an order of magnitude compared to long-term cliff retreat rates for the past ~500 years. Furthermore, these projected cliff retreat rates are unprecedented in the last 3–5 millennia for both sites. We find cliff retreat to be driven primarily by wave erosion and strongly linked to the rate of SLR, suggesting that climate change will have a direct impact on risk associated with coastal hazards in the coming century and beyond, even on historically stable coastlines. Although there is evidence for controls, other than the rate of SLR on cliff retreat at other rock coast sites^21^, these findings challenge conventional coastal management policies, in which rock coasts are considered stable environments compared to sandy coastlines^5^. This study provides clear evidence that rock coasts should be included in future planning for climate change response and, importantly, we cannot use historical rates to assess the risk associated with rock coasts because climate change will transform the currently observed, stable behaviour of these globally ubiquitous coastlines.

## Methods

### Quantifying recent cliff retreat rate

Rates of cliff retreat between ~1886 and 2015 were quantified using the Digital Shoreline Analysis System (DSAS) 5.0^58^ for ~2 km of coastline at each site encompassing the field site locations where samples for CRN analysis were taken (Supplementary Fig. 7; Supplementary Fig. 8). A length of ~2 km of coastline was chosen to capture alongshore variability into our historical cliff retreat rate calculations. The historic cliff positions were digitally mapped using georeferenced historical Ordnance Survey (OS) maps^59^ and the present-day cliff positions were mapped from a digital surface model (DSM) generated by structure-from-motion techniques using aerial photographs collected by an unmanned aerial vehicle (UAV), aerial photographs, and light detection and ranging (LiDAR) data^60^. A reference baseline was constructed from a smoothed onshore (1 m) contour line and a transect feature class was produced by projecting transects onshore and offshore, perpendicular to the baseline at 5 m intervals so that each transect intersected with both the historical and present-day cliff lines. The rate of cliff line position change was calculated by dividing the distance between the historic and modern cliff lines for each transect by the time that elapsed between the two cliff line observations. The retreat rate uncertainty value is calculated by propagating the uncertainty of the modern and historical cliff positions in quadrature and dividing by the time elapsed between the two cliff positions^58,61^. The overall uncertainty of the modern and historic cliff line positions includes uncertainties associated with the georeferencing, digitising and positional accuracy of all the imagery and maps used (Supplementary Table 4).

### CRN sample collection

Shore platform samples of fine- to medium-grained sandstone were collected along a cliff-line normal transect during spring tides to maximise platform width in August 2017 at Bideford and Scalby and August 2018 at Bideford. The return to the field site at Bideford was to extend the transect further offshore at a lower tide. At Bideford, because bedding is steeply dipping and strikes roughly perpendicular to the cliff face, the sample transect was able to follow along a 20–80 cm thick and continuous bed of fine sandstone. At Scalby, because the sandstone beds are shallowly dipping and interrupted by faults that offset beds, the sample transect covered two separate fine-grained sandstone beds. At both Bideford and Scalby, ~2 kg sandstone samples at ~10 m intervals along the transects were taken from topographic highs with a flat upper surface using a circular saw and chisel to ensure a consistent sampling depth of ~5 cm. A sample from the cliff base or sea cave was also taken to correct for inherited, muogenic-produced ^10^Be (the ^10^Be concentration produced deep in the subsurface and present in the rock before the platform was exposed to spallation interactions at or near Earth’s surface). High-accuracy (1–100 cm) Global Navigation Satellite System (GNSS) coordinates and elevation data were taken at each sample point using a Trimble Geo 7X and antenna. A laser range finder was used to measure ~10 m intervals between sample points.

### CRN sample processing

The shore platform samples were prepared using isotope dilution chemistry based on revised techniques of standard procedures^62–64^ at the CosmIC laboratory at Imperial College London. Sample preparation to separate and purify the quartz from the rock was followed by isotope dilution with a low background carrier solution to quantitatively isolate the Be and Al into a suitable form for isotopic ratio analysis by accelerator mass spectrometry (AMS)^65^. Firstly, the samples were washed, crushed and sieved to an appropriate grain size (106–150 µm) and magnetically separated to remove ferrous minerals, before undergoing a 6 N hydrochloric acid and peroxide leach to dissolve soluble material (e.g., carbonates and iron oxides) and remove organic compounds. Pure quartz was then obtained after 3× ≃1% nitric and hydrofluoric acid (HNO^3^-HF) etchings in hot ultrasonic baths. Partial etching eliminated atmospheric ^10^Be and ^26^Al because the outer part of the grain and adsorbed nuclides were removed, as well as the other more soluble minerals (e.g., feldspar). The purity of quartz was tested and verified using an Agilent 5100 SVDV inductively coupled plasma optical emission spectrometer (ICP-OES). A known mass of low-background Be carrier solution (^10^Be/^9^Be ≃ 2 × 10^−16^) with a concentration of 759.44 ± 9 ppm was then added to each quartz sample. An Al carrier solution with a concentration of 1000 ppm was added to all Scalby blank samples and rock sample SY17. The samples were dissolved in concentrated HF and HNO_3_ and the total amount of Al (native + carrier) present in the sample was calculated from ICP-OES measurements of an aliquot taken from each dissolved sample solution. Dissolved samples were then evaporated, fumed with perchloric acid to remove residual fluoride and convert to chloride form, and passed through 2 ml anion (Dowex 1 × 8 200–400 mesh resin, 1.2 meq mL^−1^) and 20 ml cation (Dowex 50WX8 200–400 mesh resin, 1.7 meq mL^−1^) exchange columns to isolate the Be and Al. Be purity and high yield was verified with ICP-OES analyses of cation column fractions. Be and Al hydroxide was precipitating using ammonia hydroxide at pH ~8, washed in 18 megohm water, and then ignited at 750 °C to convert to oxide form. BeO was mixed with Nb, and Al_2_O_3_ was mixed with Ag, at a 1:1 molar ratio^66^ to ensure good electrical and thermal conduction^67^ and was packed into copper cathodes ready for AMS analysis. Process blanks (containing ~250 µg ^9^Be and ~2500 µg ^27^Al carrier only) were subjected to the same chemical procedures as the samples^68^ to allow for quantification of background number of ^10^Be or ^26^Al atoms introduced during sample preparation.

The ^10^Be/^9^Be and ^26^Al/^27^Al analyses by AMS were conducted at the Australian Nuclear Science and Technology Organisation (ANSTO) using the 6 MV Sirius tandem accelerator^69^. AMS analysis allows a ^10^Be/^9^Be and ^26^Al/^27^Al ratio to be measured at extremely high sensitivities by removing isobaric, including molecular, interferences^67^. Measured ^10^Be concentrations were normalised to the KN-5-3 standard with a reference value for ^10^Be/^9^Be ratio of 6.320 × 10^−12^ (*t*_1/2_ = 1.36 Ma,^70^). Measured ^26^Al concentrations were normalised to Al standard KN 01-4-2 with a reference ^26^Al/^27^Al ratio of 3.096 × 10^−11 (^^71^^)^.

To determine the CRN concentrations produced during shore platform development, first, measured concentrations were calculated from the AMS-measured ratios and are corrected for chemistry background ^10^Be and ^26^Al concentrations using the process blank samples with uncertainties in the sample and background propagated in quadrature. Second, concentrations were also corrected for inherited CRNs using the ‘shielded’ cliff samples by subtracting the background-corrected concentration of these shielded samples from the background-corrected concentration of platform samples and again propagating the uncertainties in both concentrations in quadrature. Correcting concentrations using the shielded samples (‘inheritance-corrected concentrations’) corrects for any muogenic-produced CRNs present in the rock before spallogenic production becomes dominant, i.e., where spallation reactions (^10^Be produced by exposure to secondary cosmic-ray neutrons) dominate over muon-produced ^10^Be in the upper few metres of the Earth’s surface. See Supplementary Tables 1–3 for CRN concentration calculations.

### Topographic profile generation

Aerial imagery was collected with a DJI Phantom 4 Pro to produce a high-quality DSM of the cliff and platform using Pix4D mapper software^72^. At each site, 6 ground control points, located using high-precision GNSS coordinates, were used in the field to improve the quality of geo-location and orthorectification achieved by the software. A topographic swath profile used to calibrate the model, with a swath width of 10 m, was extracted from the DSM along a cross-shore transect at the location that CRN samples were collected.

### Modelling

Methodologies associated with the multi-objective optimisation approach are explained in detail in Shadrick et al.,^38^ and an overview provided here. The numerical model couples an exploratory rock coast evolution model^39^ and a dynamic model for shore platform evolution and ^10^Be production^40^ to model rock coast erosion and ^10^Be production simultaneously. The model applies a dynamic form of coastal evolution that allows transient shore platform development and cliff retreat across millennial timescales. Simulated wave erosion expresses wave hydraulic and mechanical properties as wave assailing force, consisting of wave height, modified by an exponential decay function to approximate wave breaking energy dissipation, following established rock coast evolution models^25,41,73^. Cliff erosion is necessarily simplified given the millennial timescale of modelled cliff erosion, so that cliff retreat is exclusively driven by wave erosion at the cliff foot and detailed processes acting directly to the cliff face, such as subaerial weathering and saturation from groundwater, are not represented. The model space is categorised into a gridded cell framework. Erosion of the shore platform and cliff is achieved once wave assailing force exceeds a material resistance value assigned to the rock material of a given cell. Subaerial inter-tidal weathering is also simulated and works to lower the resistance of the rock material value^39^. The concentration of ^10^Be is updated across the shore platform at every annual time step. Both spallation-produced ^10^Be at the surface and muon-produced ^10^Be at depth are modelled. Cliff retreat exposes new shore platform material to ^10^Be spallation production. Exposure to ^10^Be production is modulated through time due to the evolving geomorphology due to cliff retreat, platform lowering, water cover (including tidal variation and SLR) and topographic shielding^33,40^. These factors combined usually predict a ‘humped’ ^10^Be concentration profile across the shore platform, where the magnitude of the hump is inversely proportional to the rate of cliff retreat^1,33^.

For each site, two model outputs, a topographic profile and ^10^Be concentration profile, were optimised simultaneously using multi-objective optimisation with Queso Bayesian calibration library^74^ within Dakota optimisation environment^75^. A 10,000 iteration Metropolis-Hastings MCMC^76^ simulation was used to refine a set of model input parameters that produce model outputs that best match the measured profile data. This was achieved by minimising the negative log-likelihood score from an equally weighted objective function that combines both topographic profile residuals and ^10^Be concentration residuals between modelled and measured results. See Shadrick et al.^38^ for full explanation of how this objective function is formulated and applied within the Dakota environment. Free parameters chosen to vary within the MCMC simulation were wave erodibility by means of wave height decay rate (*y*), material resistance (*F*_*R*_) and maximum intertidal weathering rate (*K*). Free parameter selection was informed by previous investigations that found these variables had the greatest influence on rock coast evolution and the relative importance of wave or weathering-driven erosion^27^. The broad exploratory parameter space was informed by both modelling-based and field-based investigations^27,39,73,77^. Altering the value of wave height decay rate (*y*, m^−1^) results in varied erosion achievable by breaking and broken waves. A greater value for *y* means wave energy will dissipate rapidly, so less erosion is achieved, while a lesser value for *y* results in slow wave height decay, allowing energy to propagate further across the shore platform, resulting in more erosion. A single value for material resistance (*F*_*R*_, kg m^−2^ yr^−1^) is highly simplified and incorporates mechanical, geological and structural controls into a single value^39^. Maximum weathering rate (*K*, kg m^−2^ yr^−1^) occurs at the mean high water neap tidal level (MHWN) with the distribution of intertidal weathering defined by a weathering efficacy function^50^. The range of *K* explored encompasses a parameter space where negligible intertidal weathering and where weathering rate equal to the material resistance (*F*_*R*_) can be replicated in the MCMC simulations. Uncertainty on best fit results were defined by the 16% and 84% confidence intervals of likelihood-weighted posterior distributions of accepted sample positions^75^.

For modelling past cliff retreat, site-specific sea-level histories from 8000 years BP to present were derived from a glacial isostatic adjustment (GIA) model^44^. A fixed mean spring tidal range, based on tidal gauge measurements^78^, was used as a model input. Future forecasts of cliff retreat in response to accelerated RSL rise use the best-fit parameter values and UKCP18 future sea-level predictions for RCP2, RCP4 and RCP8 scenarios (Supplementary Fig. 1). Uncertainties in future cliff retreat forecasts include model uncertainty and 5% and 95% confidence intervals for all RCP scenarios. The coastal evolution model was developed to simulate rock coast evolution over millennial timescales. So, the model output of retreat rates over decadal timescales for future cliff retreat forecasts, up to the year 2100, needs to be considered carefully. Two moving average windows were used; one of 1000-year period BP and a second of 100-year period up to 2100, were used to smooth individual cliff erosion events so that the long-term trend in cliff retreat rates could be identified (Fig. 3, Supplementary Fig. 5). Comparisons between the long-term trends and individual erosion events are shown (Supplementary Fig. 6). The cell resolution of the model was set to 10 cm and the topographic and ^10^Be concentration profile were updated every year (1-year timestamps). The spatial and temporal resolution is consistent with that used for the model optimisation for the past 8000 years. This means individual erosion events captured by the model cannot be less than 10 cm of cliff retreat. Using a moving average window averages the cliff retreat of all erosion events within the moving window timescale so that average cliff retreat rates of less than 10 cm y^−1^ could be identified.

### Supplementary information

Supplementary Information

## Data Availability

The input data and calculations generated in this study are provided in the Supplementary Information.

## Code availability

The coupled model, model optimisation code and documentation are publicly available online (10.5281/zenodo.5645478).
