# Supplementary material for: Sea-level rise will likely accelerate rock coast cliff retreat rates
Source: Nat Commun. 2022 Nov 18;13:7005. doi: 10.1038/s41467-022-34386-3 (PMC9674839; doi:10.1038/s41467-022-34386-3)
Supplement: Supplementary file 1 — Supplementary Information [file 41467_2022_34386_MOESM1_ESM.pdf]

Supplementary Information

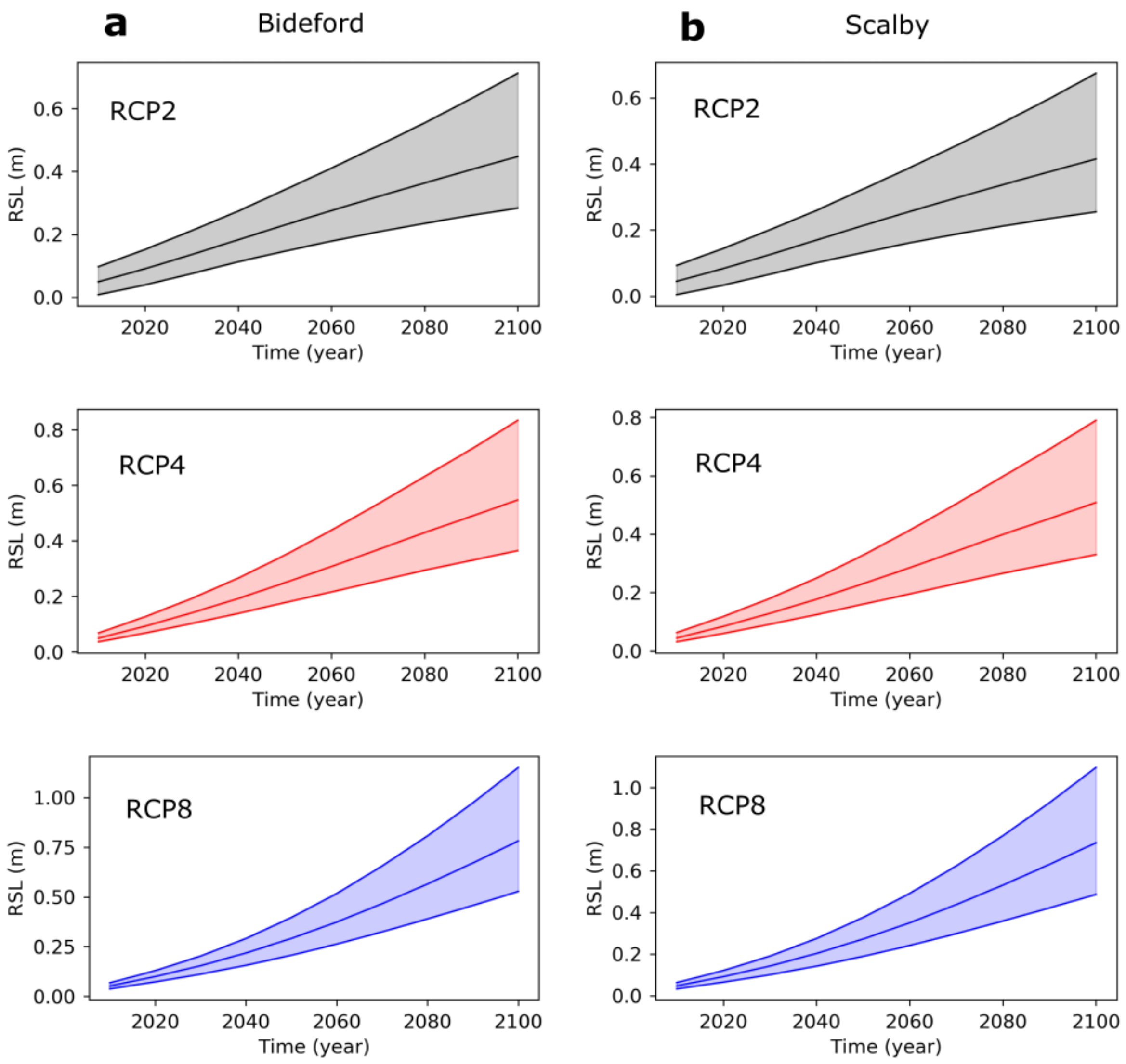

Supplementary Fig 1: UKCP18 RCP2, RCP4 and RCP8 sea level scenarios from 2000 to 2100. (a) Bideford **(b)** Scalby. Solid lines show 5th, 50th and 95th percentiles.

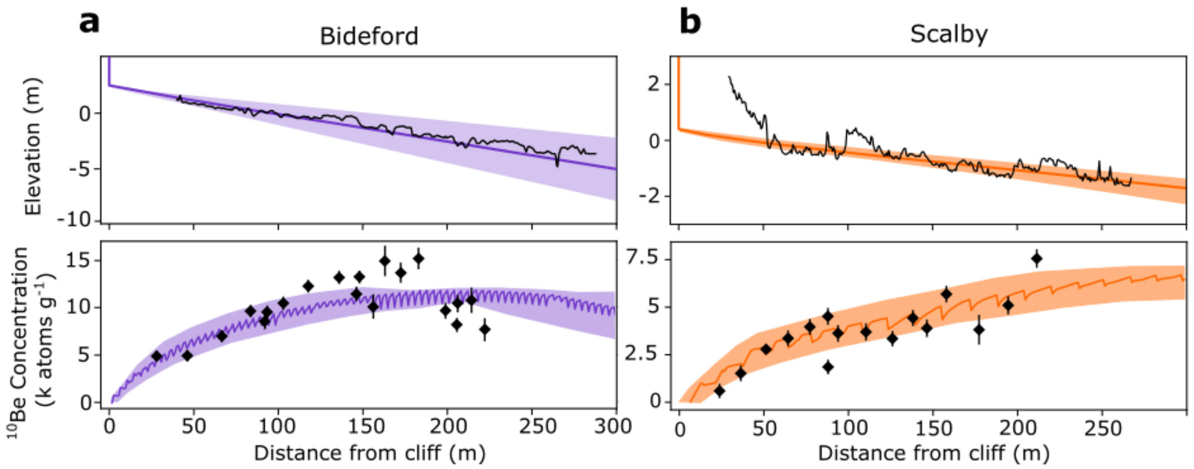

Supplementary Fig. 2: Best-fit output model topography and ^{10}Be concentrations. (**a**) Best-fit results for Bideford. (**b**) Best-fit results for Scalby. Coloured, dark lines show best-fit model results and shaded area show 16%–84% confidence interval. Black line shows measured topographic swath profile to compare to model output. Scatter show measured, chemistry background and inheritance corrected ^{10}Be concentrations to compare to model output.

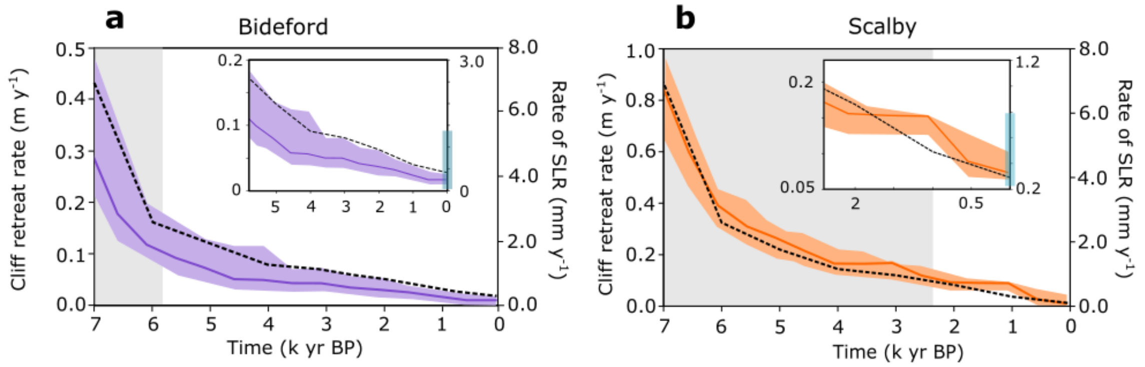

Supplementary Fig. 3: A time series of cliff retreat rates for the past 7000 years. Cliff retreat rates (m y^{-1}) are calculated for Bideford (**a**) and Scalby (**b**) from modelled cliff position from 7000 years (BP) to present-day. Inset plots show the unshaded area that corresponds to the distance across the shore platform over which measured data were analyzed. Modern cliff retreat rates (shaded blue) are also shown in the inset plots at the present-day time stamp. The corresponding rate of SLR (mm y^{-1}) is shown by the black dashed line.

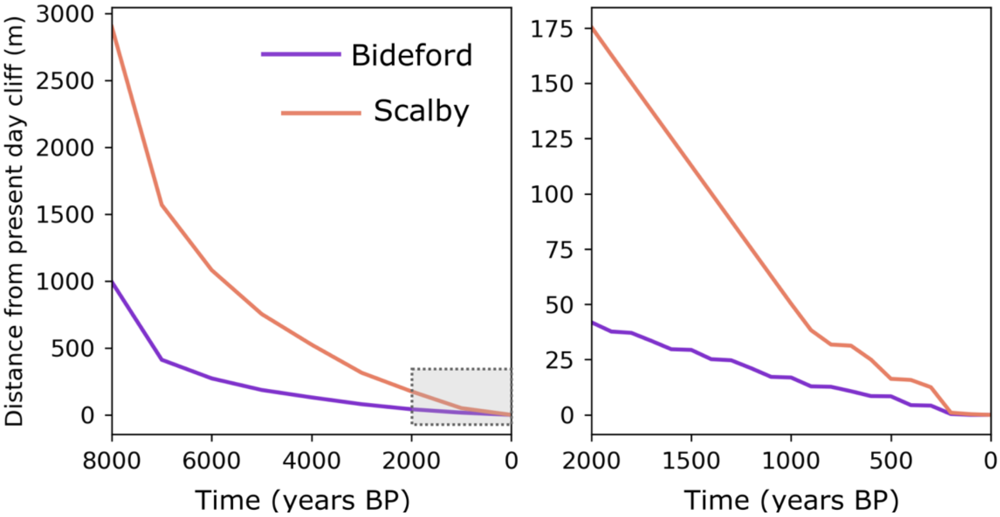

Supplementary Fig. 4: Distance of cliff position through time. Cliff position normalised to the position of the present-day cliff position at 0 m from 8,000 years BP to present-day at 0 years BP.

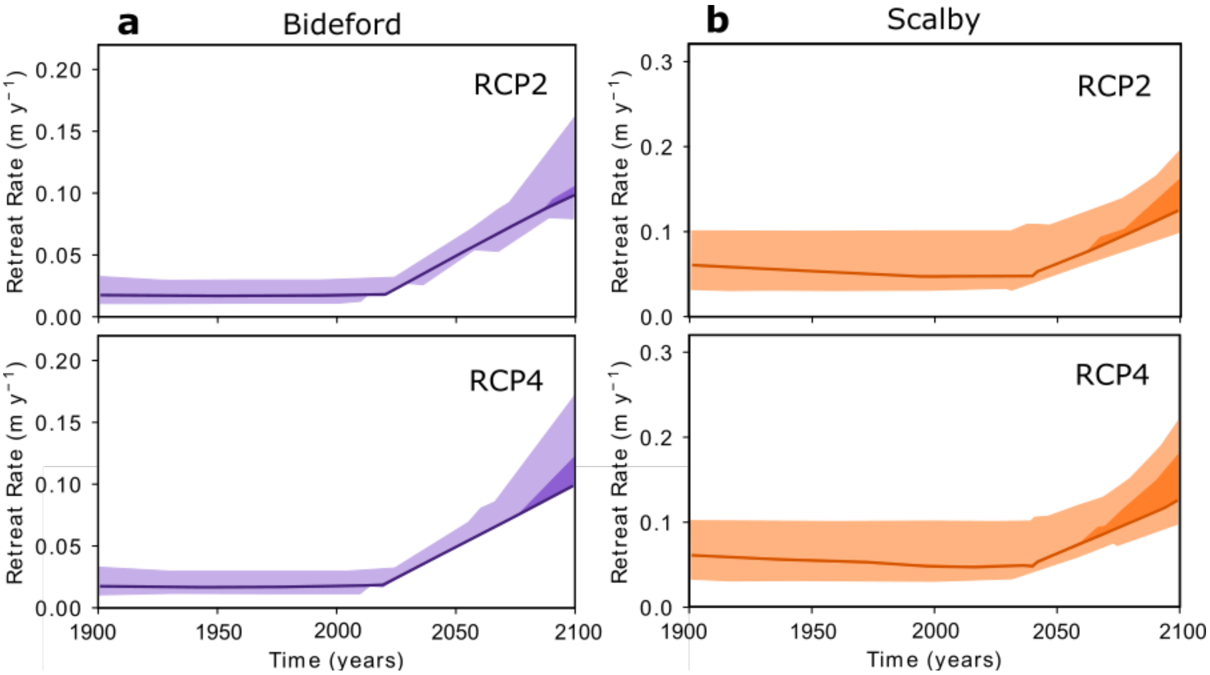

Supplementary Fig. 5: Cliff retreat rates (m y^{-1}) for the years 1900 to 2100 for RCP2 and RCP4 scenarios. (**a**) RCP2 and RCP4 future forecasts of cliff retreat rates at Bideford. (**b**) RCP2 and RCP4 future forecasts of cliff retreat rates at Scalby.

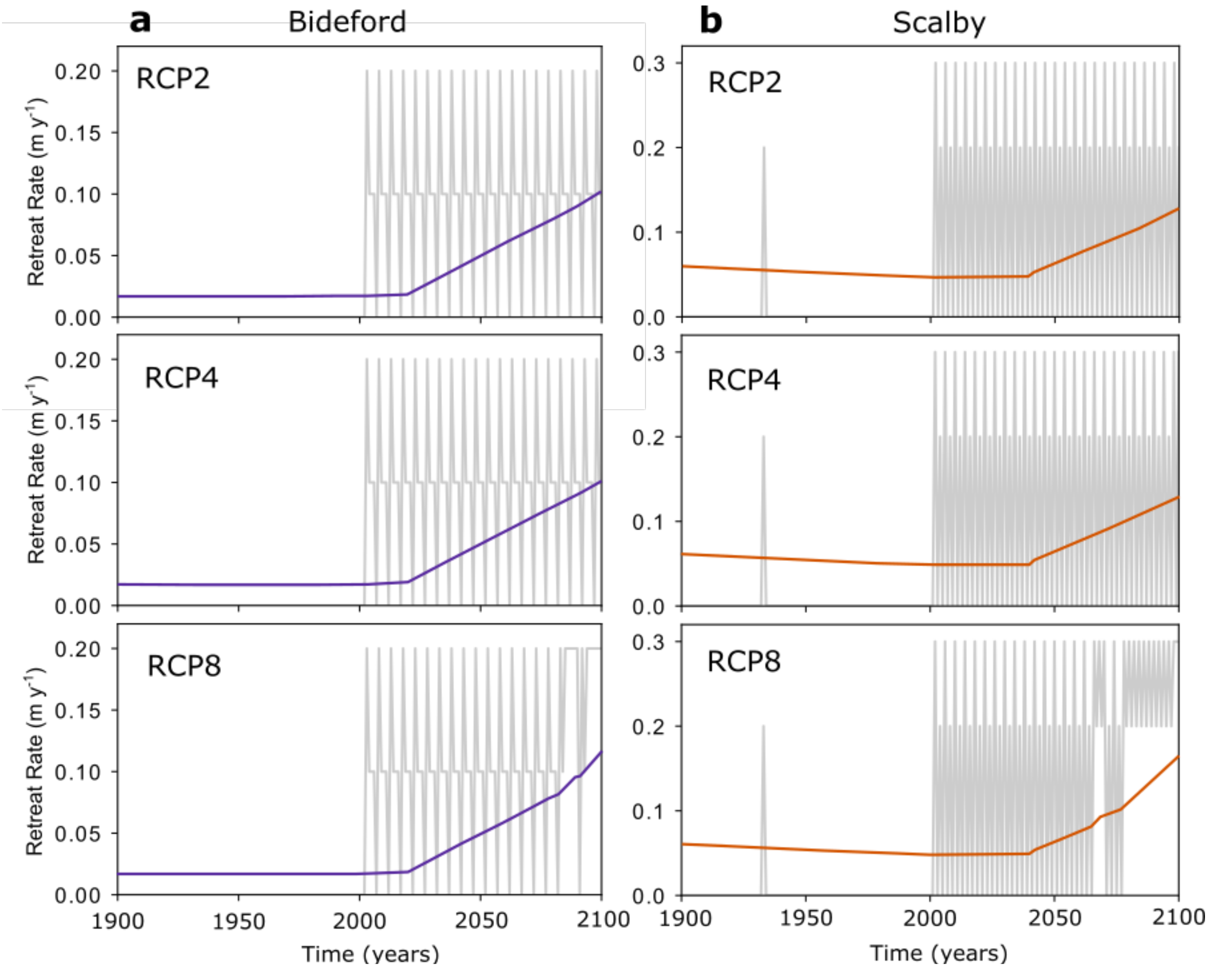

Supplementary Fig. 6: Long-term trends in cliff retreat rates and individual erosion events for the years 1900 to 2100. (**a**) Cliff retreat rate for best-fit model runs and median RCP2, RCP4 and RCP8 scenarios for Bideford. (**b**). Cliff retreat rate for best-fit model runs and median RCP2, RCP4 and RCP8 scenarios for Scalby. Grey lines show the 1-year averaged cliff retreat rates and coloured lines show the 1000-year moving average (before year 2000) and 100-year moving average (after year 2000) trend in cliff retreat rates.

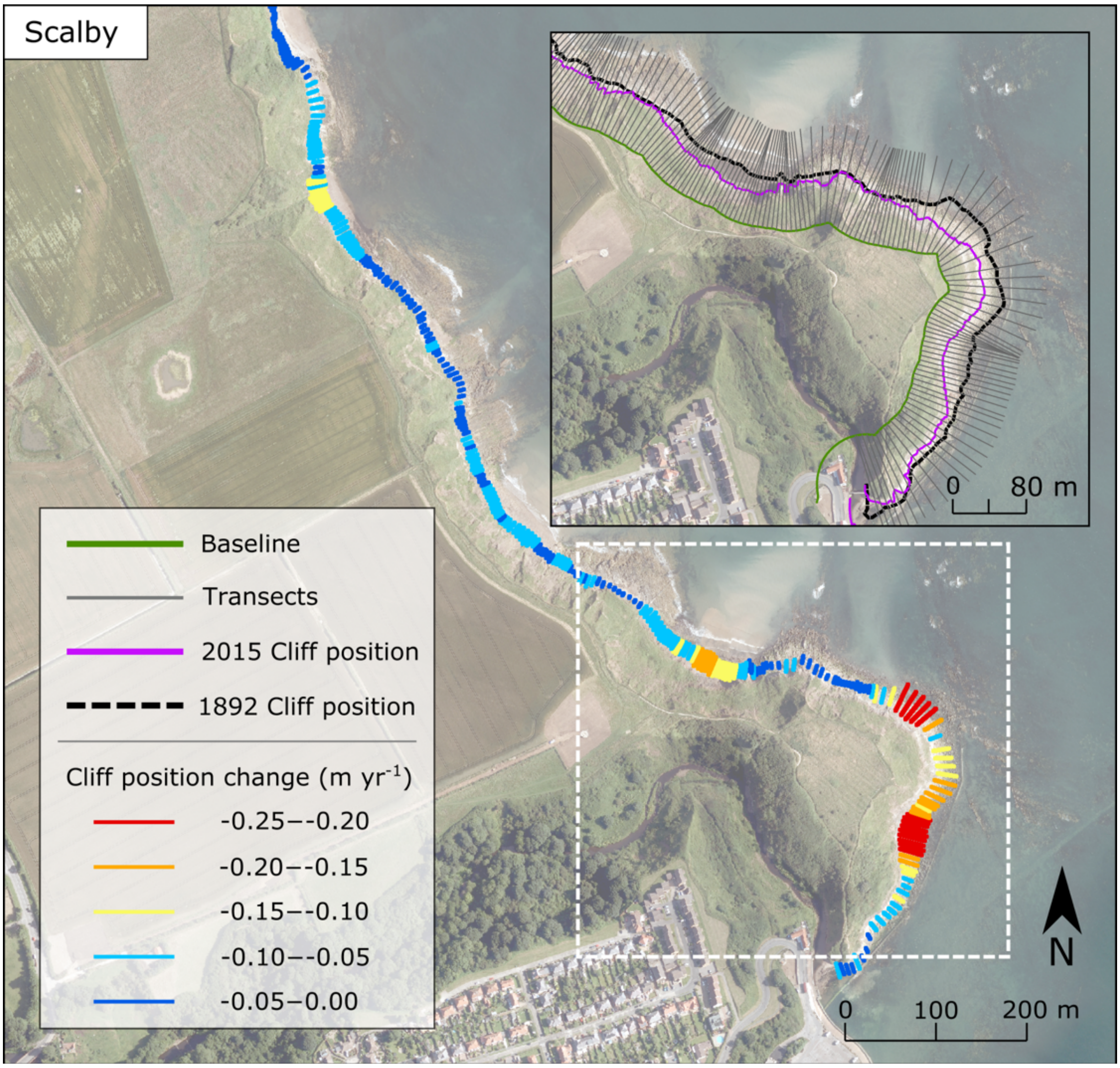

Supplementary Fig. 7: DSAS calculations of historical cliff retreat rates for Scalby. Historical and present-day cliff lines, transects and baseline used to calculate historical cliff retreat rates at Scalby.

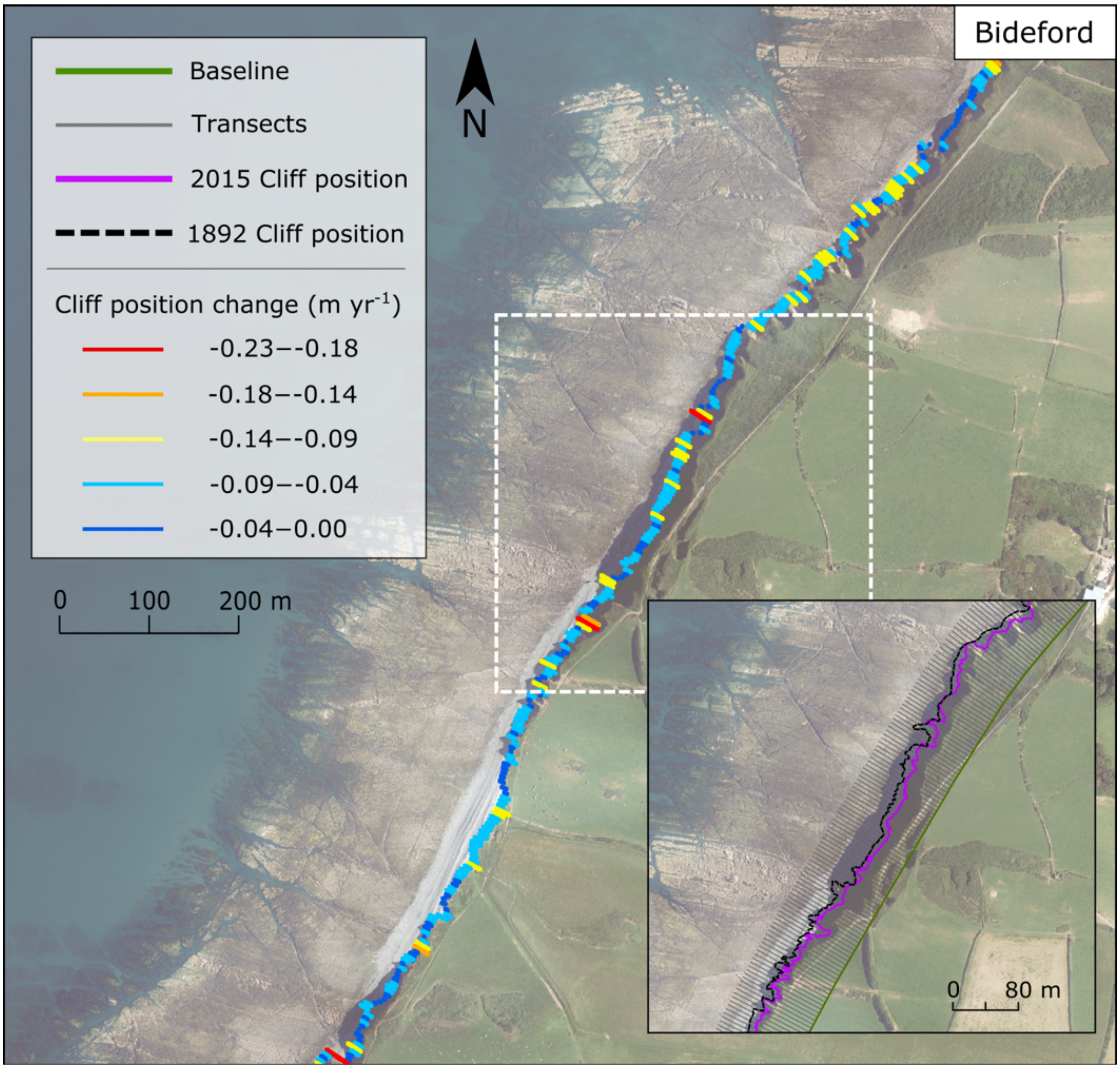

Supplementary Fig. 8: DSAS calculations of historical cliff retreat rates for Bideford. Historical and present-day cliff lines, transects and baseline used to calculate historical cliff retreat rates at Bideford.

Supplementary Table 1: ^{10}Be sample and concentration data for Bideford field site

Batch	Sample ID	Location (UTM 30U)		Distance from cliff (m)	Elevation (m)	Mass of quartz dissolved (g)	Mass of carrier added (g)*	Measured $^{10}Be/^{9}Be$ ratio (x 10^{-14})	± 1σ AMS analytical uncertainty $^{10}Be/^{9}Be$ ratio (x 10^{-14})	Background-corrected Concentration ^{10}Be (x10^3 atoms g^{-1})**	± 1σ AMS Analytical uncertainty (x 10^3 atoms g^{-1})	Inheritance corrected ^{10}Be *** (x 10^3 atoms g^{-1})	± **** (x 10^3 atoms g^{-1})
		Easting (m)	Northing (m)										
1	CB05	411280.69	5654197.60	82.9	0.84	41.434	0.334	3.05	0.118	12.25	0.49	9.24	0.66
	CB07	411246.98	5654206.81	116.4	0.09	42.614	0.335	3.79	0.125	14.49	0.50	11.88	0.67
	CB09	411218.34	5654214.09	144.6	-0.80	33.681	0.333	2.86	0.123	14.06	0.62	11.06	0.76
	CB11	411215.47	5654212.84	146.4	-0.98	38.978	0.334	3.70	0.121	15.85	0.53	12.84	0.69
	BLK050318				-		0.335	0.06	0.015				
2	CB01 (cave)	411228.72	5653989.15		-	21.453	0.334	0.60	0.049	3.85	0.52		
	CB10a	411273.74	5654204.71	92.6	0.49	38.225	0.335	2.84	0.112	12.13	0.53	9.13	0.69
	BLK140318				-		0.335	0.11	0.043				
3	CB02	411336.19	5654183.19	28.2	1.91	38.030	0.336	1.90	0.080	7.49	0.38	4.48	0.58
	CB03	411318.95	5654189.18	46.0	1.44	38.013	0.337	1.91	0.097	7.53	0.45	4.52	0.63
	CB04	411298.74	5654194.64	66.1	1.10	38.450	0.337	2.39	0.098	9.60	0.46	6.59	0.64
	CB06	411264.28	5654207.30	102.0	0.30	38.132	0.337	3.14	0.119	13.07	0.55	10.06	0.71
	CB08	411228.81	5654211.83	134.5	-0.25	32.940	0.337	3.28	0.100	15.81	0.55	12.80	0.70
	BLK120918				-		0.338	0.23	0.029				
4	CB21	411181.91	5654223.88	180.7	-2.37	24.686	0.332	2.97	0.151	17.82	1.06	14.82	1.15
	CB22	411192.96	5654222.02	170.3	-1.48	30.086	0.34	3.20	0.166	16.31	0.98	13.30	1.07
	CB23	411202.53	5654220.08	161.2	-1.19	21.132	0.332	2.56	0.190	17.56	1.55	14.55	1.61
	CB24	411209.59	5654218.62	154.4	-1.35	19.904	0.336	1.84	0.136	12.74	1.21	9.73	1.29
	CB25	411140.64	5654230.59	219.3	-2.62	12.253	0.335	1.10	0.073	10.28	1.13	7.27	1.22
	CB26	411149.54	5654230.22	211.5	-3.50	11.461	0.335	1.26	0.077	13.39	1.27	10.38	1.34
	CB27a	411158.22	5654228.96	203.5	-2.70	18.072	0.336	1.74	0.077	13.03	0.81	10.03	0.92
	CB27b	411159.14	5654229.72	203.1	-2.87	30.627	0.336	2.29	0.103	10.77	0.61	7.77	0.76
	CB28	411166.49	5654228.93	196.5	-3.61	32.048	0.336	2.66	0.13	12.27	0.72	9.27	0.85
	CB29 (cliff)	411366.28	5654178.11		-	32.886	0.336	0.94	0.077	3.00	0.44		
	CB10b	411275.02	5654204.35	91.3	0.56	30.166	0.337	2.32	0.112	11.12	0.67	8.11	0.80
	BLK270918				-		0.337	0.36	0.038				

*Carrier concentration 759.44 μg Be g^{-1}.

** Normalised to the KN-5-3 standard with an assumed $^{10}Be/^{9}Be$ ratio of 6.320 x 10^{-12} (1). Values corrected for chemistry background using batch-specific full chemistry blanks processed in each batch with errors in sample and blanks propagated in quadrature

***All CB samples were corrected for inheritance with cliff sample CB29

**** Error propagated as $\sigma_c = \sqrt{\sigma_a^2 + \sigma_b^2}$ where σ_a is the error of the measured concentration, σ_b is the error of the measured concentration used for the correction CB29

Supplementary Table 2: ^{10}Be sample and concentration data for Scalby field site

Batch	Sample ID	Location (UTM 30U)		Distance from cliff (m)	Elevation (m)	Mass of quartz dissolved (g)	Mass of carrier added (g)*	Measured ^{10}Be/^{9}Be ratio (x 10^{-14})	± 1σ AMS analytical uncertainty ^{10}Be/^{9}Be ratio (x 10^{-14})	Background-corrected Concentration ^{10}Be (x10^{3} atoms g^{-1})**	± 1σ AMS Analytical uncertainty (x 10^{3} atoms g^{-1})	Inheritance corrected ^{10}Be *** (x 10^{3} atoms g^{-1})	± **** (x 10^{3} atoms g^{-1})
		Easting (m)	Northing (m)										
	SY02	668799.11	6020464.15	144.8	0.21	35.796	0.320	1.67	0.085	7.30	0.39	3.88	0.46
	SY04	668779.64	6020476.36	124.4	-0.23	47.726	0.334	1.96	0.089	6.75	0.32	3.33	0.41
	SY07	668710.4	6020547.10	49.8	1.12	42.984	0.334	1.63	0.870	6.19	0.35	2.77	0.43
1	SY10	668745.77	6020531.14	86.3	0.56	48.569	0.334	2.33	0.110	7.93	0.37	4.51	0.44
	SY13	668764.36	6020481.03	108.8	0.29	35.668	0.335	1.55	0.079	7.10	0.38	3.69	0.46
	SY15	668849.73	6020493.97	192.9	-0.48	40.654	0.334	2.10	0.098	8.51	0.41	5.09	0.48
	SY17 (cliff)	668657.31	6020508.17		-	44.773	0.334	0.96	0.063	3.42	0.25		
	BLK050318				-		0.335	0.06	0.015				
	SY06	668696.00	6020554.48	34.8	1.02	41.169	0.334	1.31	0.070	4.93	0.34	1.51	0.42
2	SY11	668742.33	6020486.68	86.4	0.84	49.228	0.335	1.64	0.075	5.26	0.30	1.84	0.39
	BLK140318				-		0.335	0.11	0.043				
	SY01	668810.53	6020461.9	156.4	0.07	38.079	0.336	2.17	0.078	9.09	0.36	5.68	0.44
	SY03	668791.19	6020468.65	136.6	0.32	38.449	0.337	1.91	0.075	7.84	0.35	4.42	0.43
	SY05	668683.96	6020561.27	22.3	1.16	34.947	0.337	0.96	0.054	4.00	0.29	0.58	0.38
	SY08	668723.12	6020543.00	62.8	0.41	34.748	0.337	1.52	0.063	6.77	0.33	3.35	0.41
3	SY09	668735.62	6020538.00	75.6	0.55	38.129	0.337	1.78	0.072	7.37	0.34	3.95	0.42
	SY12	668748.06	6020484.77	92.3	0.51	38.065	0.337	1.71	0.082	7.04	0.38	3.62	0.46
	SY14	668867.17	6020499.85	209.8	-0.98	38.011	0.338	2.58	0.090	10.97	0.42	7.55	0.48
	SY16	668831.57	6020483.06	175.7	-0.21	9.973	0.337	0.56	0.037	7.22	0.74	3.80	0.78
	BLK240918				-		0.338	0.14	0.023				

*Carrier concentration 759.44 μg Be g^{-1}.

** Normalised to the KN-5-3 standard with an assumed ^{10}Be/^{9}Be ratio of 6.320 x 10^{-12} [(1)]. Values corrected for chemistry background using batch-specific full chemistry blanks processed in each batch with errors in sample and blanks propagated in quadrature

***All SY samples were corrected for inheritance with cliff sample SY17

**** Error propagated as $\sigma_c = \sqrt{\sigma_a^2 + \sigma_b^2}$ where σ_a is the error of the measured concentration, σ_b is the error of the measured concentration used for the correction SY17

Supplementary Table 3: ^{26}Al sample and concentration data for Scalby field site

**Batch**	**Sample ID**	**Location (UTM 30U)**		**Distance from cliff (m)**	**Elevation (m)**	**Mass of quartz dissolved (g)**	**Total ^{27}Al in dissolution solution (ICP) (µg)**	**Measured ^{26}Al/^{27}Al ratio (x 10^{-14})**	**± 1σ AMS analytical uncertainty ^{26}Al/^{27}Al ratio (x 10^{-14})**	**Background-corrected Concentration ^{26}Al (x10^4 atoms g^{-1})****	**± 1σ AMS Analytical uncertainty (x 10^4 atoms g^{-1})**	**Inheritance corrected ^{26}Al *** (x 10^4 atoms g^{-1})**	**± **** (x 10^4 atoms g^{-1})**
		Easting (m)	Northing (m)										
1	SY02	668799.11	6020464.15	144.8	0.21	35.796	3028	3.11	0.210	5.80	0.40	3.59	0.46
	SY04	668779.64	6020476.36	124.4	-0.23	47.726	4649	1.62	0.270	3.44	0.59	1.23	0.63
	SY07	668710.40	6020547.10	49.8	1.12	42.984	3422	2.18	0.230	3.81	0.41	1.59	0.47
	SY10	668745.77	6020531.14	86.3	0.56	48.569	4345	2.78	0.208	5.48	0.42	3.27	0.48
	SY13	668764.36	6020481.03	108.8	0.29	35.668	2974	2.83	0.218	5.19	0.41	2.98	0.47
	SY15	668849.73	6020493.97	192.9	-0.48	40.654	3248	3.34	0.243	5.89	0.44	3.67	0.49
	SY17 (cliff)	668657.31	6020508.17		-	44.773	2450	1.85	0.184	2.22	0.23		
	BLK050318				-		2591	0.04	0.025				
2	SY06	668696.00	6020554.48	34.8	1.02	41.169	3229	2.07	0.240	3.51	0.42	1.29	0.48
	SY11	668742.33	6020486.68	86.4	0.84	49.228	3197	3.05	0.282	4.33	0.41	2.11	0.47
	BLK140318				-		2641	0.06	0.036				
3	SY01	668810.53	6020461.90	156.4	0.07	38.079	3646	2.79	0.130	5.64	0.28	3.42	0.36
	SY03	668791.19	6020468.65	136.6	0.32	38.449	3787	2.63	0.117	5.46	0.26	3.24	0.35
	SY05	668683.96	6020561.27	22.3	1.16	34.947	2781	1.65	0.090	2.68	0.17	4.60	0.28
	SY08	668723.12	6020543.00	62.8	0.41	34.748	3199	2.57	0.115	4.98	0.24	2.76	0.33
	SY09	668735.62	6020538.00	75.6	0.55	38.129	3224	2.79	0.118	4.99	0.23	2.78	0.32
	SY12	668748.06	6020484.77	92.3	0.51	38.065	3674	2.27	0.109	4.58	0.24	2.36	0.33
	SY14	668867.17	6020499.85	209.8	-0.98	38.011	4031	3.11	0.144	7.01	0.35	4.79	0.41
	SY16	668831.57	6020483.06	175.7	-0.21	9.973	952	2.88	0.220	5.82	0.47	3.60	0.52
	BLK240918				-		2738	0.15	0.026				

** Normalised to the KN 01–4–2 standard with a nominal ^{26}Al/^{27}Al ratio of 3.096 x 10^{-11} (2). Values corrected for chemistry background using batch-specific full chemistry blanks processed in each batch with errors in sample and blanks propagated in quadrature

***All SY samples were corrected for inheritance with cliff sample SY17

**** Error propagated as $\sigma_c = \sqrt{\sigma_a^2 + \sigma_b^2}$ where σ_a is the error of the measured concentration, σ_b is the error of the measured concentration used for the correction SY17

Supplementary Table 4. Uncertainty calculations for historical cliff retreat rates. Georeferencing value from RSM error in ArcGIS, OS map positional and survey error as suggested by Dornbusch et al,[3]. Aerial imagery positional error, supplied by Channel Coast Observatory.

Measurement uncertainties (m)	Bideford		Scalby	
	1886 cliff	2015 cliff	1892 cliff	2015 cliff
Georeferencing	0.04	0	0.04	0
Digitizing	1	0.5	1	0.5
OS map positional error	5	0	5	0
Survey error	1.1	0	1.1	0
Aerial positional error	0	0.3	0	0.3
Orthophotography positional error	0	0.1	0	0.1
Total positional accuracy (m)	5.2	0.6	5.2	0.6
Annualized retreat rate uncertainty (cm/year)	4.1		4.3	

Supplementary References

1. Nishiizumi, K. *et al.* Absolute calibration of ^{10}Be AMS standards. *Nucl. Instrum. Methods Phys. Res. Sect. B Beam Interact. Mater. At.* **258**, 403–413 (2007).
2. Nishiizumi, K. Preparation of ^{26}Al AMS standards. *Nucl. Instrum. Methods Phys. Res. Sect. B Beam Interact. Mater. At.* **223–224**, 388–392 (2004).
3. Dornbusch, U., Robinson, D. A., Moses, C., Williams, R. & Costa, S. Retreat of Chalk cliffs in the eastern English Channel during the last century. *J. Maps* **2**, 71–78 (2006).
